# Toward an Extensible Regulatory Framework for N-of-1 to N-of-Few Personalized RNA Therapy Design

**DOI:** 10.1007/s43441-025-00752-8

**Published:** 2025-02-27

**Authors:** Mélissa Bou-Jaoudeh, Gabriele Piaton-Breda, Florian Pereme, Stephen Gilbert

**Affiliations:** 1 ProductLife Group, Courbevoie, France; 2 Carl Gustav Carus University Hospital Dresden, Dresden University of Technology, Dresden, Germany; 3 Else Kröner Fresenius Center for Digital Health, TUD Dresden University of Technology, Dresden, Germany

**Keywords:** Individualized RNA therapeutics, Rare diseases, Regulatory framework, Design envelope, Artificial intelligence in drug development, Predetermined Change Control Plan (PCCP)

## Abstract

The emergence of personalized RNA therapeutics, tailored to individual patients' genetic profiles, offers new hope for treating both common and rare diseases. This review explores regulatory aspects of N-of-1 and N-of-few approaches, providing promising treatments for ultra- or nano-rare diseases that lack established therapies. These diseases present unique challenges, as patients may represent the sole individual or a small group worldwide with a specific mutation, necessitating personalized approaches to treatment development, validation, and approval. While progress is promising, the regulatory landscape remains nascent, raising challenges in ensuring safety and industry sustainability. Artificial intelligence (AI) and automated systems, coupled with real-world evidence (RWE) monitoring, offer significant potential to address these challenges by optimizing development, manufacturing, and regulatory compliance. Drawing parallels from other regulatory domains, this review presents a design envelope framework, integrated with AI tools, to streamline the approval process and enhance the adaptability of RNA-based treatments. Case studies of individualized RNA-based treatments highlight successes and setbacks, underscoring the need for regulatory alignment. Collaborative efforts from stakeholders and regulatory authorities are essential to refine this framework for real-world application. Overall, this review emphasizes the transformative potential of personalized RNA therapeutics in advancing precision medicine.

## Main

In the ever-evolving landscape of medicine, personalized RNA therapeutics stand out as an innovation of recognized high implementation potential. RNA editing technologies [1], including single-base editing and RNA exon editing, [2, 3] hold promise for correcting specific RNA sequences associated with genetic disorders or cancers [4]. Unlike permanent genetic alterations induced by CRISPR (one of which recently was authorized for sickle cell disease treatment in the UK [5]), RNA editing offers a transient approach, potentially enhancing safety.

Antisense oligonucleotides (ASOs) represent versatile tools for precisely modulating gene expression at the RNA level [6]. These chemically modified DNA or RNA-like structures are designed to selectively bind messenger RNA (mRNA) molecules, offering targeted interventions without altering DNA. ASOs are particularly adept at addressing rare genetic mutations, often in a mutation-specific or even individual-specific manner, minimizing off-target effects. Similarly, small interfering RNA (siRNA) therapies, harnessing the natural RNA interference (RNAi) pathway, present a potent means of silencing disease-causing genes at the RNA level by targeting and degrading complementary mRNA molecules.

N-of-1 or bespoke therapies are tailored to the unique genetic characteristics of individual patients. Initially designed for a single patient, these therapies can also extend to small populations (N-of-few) where various mutations within the same gene produce comparable effects on the gene product. These approaches are currently most successful in diseases where targeted tissue access, such as in the brain or eye, is relatively straightforward compared to more challenging tissues like muscle. This adaptable strategy enables personalized medicine, taking account of the unique genetic and molecular makeup of each patient, such as gene deletions and rearrangements., It facilitates the development of targeted solutions for a wide range of medical conditions while minimizing side effects and optimizing outcomes. [7]

The regulatory landscape for these therapies remains complex. We argue that a more structured framework is necessary to maximize the potential of this approach while also ensuring safety, efficacy, and patient welfare. The U.S. Food and Drug Administration (FDA) has provided specific guidance for ASOs, making them one of the few technologies with tailored regulatory advice for individualized drug-based therapies. This guidance includes preclinical, manufacturing, and clinical administration protocols tailored to patients with nano-rare conditions [8–11], facilitating the rapid development of individualized RNA treatments. In contrast, the European Medicines Agency (EMA) has not issued specific guidelines for N-of-1 treatments.

In this Perspective, we examine the regulatory, scientific, and industrial challenges unique to developing N-of-1 and N-of-few RNA therapies. These highly personalized treatments, designed for individuals or small groups of patients with ultra-rare or nano-rare genetic conditions, require specialized frameworks, to ensure their safe and effective application. The adaptability of N-of-1 therapies to individual patients is key to creating an optimized regulatory pathway. We also draw on regulatory precedents from other fields and emphasize the integration of AI into different phases of treatment development, proposing a comprehensive framework for the design, manufacturing, and implementation of N-of-1 or N-of-few therapies. This includes addressing diverse (epi)genetic profiles in these rare conditions.

## Considerations and Regulatory Challenges in N-of-1 and N-of-Few Therapies

In the US, rare diseases are defined as those affecting fewer than 200,000 people, while in the EU, the threshold is no more than 1 person in 2000 [12]. With approximately 7000 rare diseases identified (Orphanet; Global Genes), about 95% lack authorized treatments, highlighting a significant unmet medical need [13]. Although terms like “ultra-rare” and “nano-rare” have been used informally, there are no formal definitions by the EMA or FDA. Ultra-rare diseases typically affect fewer than 1 in 50,000 people [14], or fewer than a few hundred individuals worldwide. Nano-rare conditions, often affecting fewer than 100 individuals globally, typically require highly personalized approaches such as N-of-1 therapies, where treatments are tailored specifically for individual patients*.* [15, 16]

While terms like “N-of-1,” “N-of-few,” or “N-of-1 + ” [17] lack universal regulatory definitions, they generally describe approaches focusing on treating individual patients or small groups of patients rather than large populations, and are commonly applied in personalized medicine. The “N” represents the number of individuals involved in the treatment. These terms should not be mistaken for “N-of-1 trials”, which are a specific type of cross-over study design. N-of-1 treatments are highly personalized, making standard trial designs impractical due to the small sample sizes. This creates challenges in achieving statistically significant results required for regulatory approval. These challenges are further compounded by the need for individualized clinical outcome assessments (COAs) and natural history data tailored to the patient’s unique genotype-phenotype. This approach aligns with recent frameworks proposed for maintaining scientific rigor in N-of-1 trials. [18]

In the US, N-of-1 treatments require a sponsor-investigator to submit a Research Investigational New Drug (IND) application to the FDA [8]. The Research IND pathway is favored over the expanded access program, also known as “Compassionate Use Program,” which provides investigational drugs to patients with serious or life-threatening conditions outside of clinical trials, bypassing specific trial criteria or addressing comorbidities. The primary distinction between the two pathways lies in the administrative processes: Form 1571 is used for Research INDs, whereas the more streamlined Form 3926 is required for expanded access [19, 20]. Regulatory review for Research INDs typically spans 30 days, though the timeframe can be expedited in cases of extreme severity. In contrast, expanded access requests can be processed in as little as few hours or up to 30 days depending on the urgency of the situation [21]. Notably, a full Institutional Review Board (IRB) is required for Research INDs [20], whereas administrative review or concurrence from an IRB chairperson suffices for expanded access. Additionally, the submission of a Research IND indicates non-commercial intent from the sponsor. The FDA has issued draft guidance documents outlining criteria for the development of individualized ASO therapies, typically for one or two patients [8–10]. These guidelines specify that these products should belong to well-characterized chemical classes with substantial clinical and nonclinical experience. Available guidance is limited to unconjugated ASO, manufactured conventionally, including formulations like single-stranded phosphorothioate or mixed phosphorothioate/phosphodiester 2-methoxyethyl substituted oligonucleotides (by systemic or intrathecal route) and phosphorodiamidate morpholino oligonucleotides (by systemic route).

In Europe, unlike in the US, no IND application is required for N-of-1 therapies. Instead, individual patients can be treated through named-patient programs (NPPs) [22] under Article 5(1) of Directive (EC) 2001/83, which allows provision of unlicensed medicinal products under the direct responsibility of an authorized healthcare professional. Physicians request supply of the unauthorized medicine directly from the manufacturer, with approval from the locally responsible ethics committee required, typically the institutional ethics committee. Although communication with regulators is advisable for guidance on safety and toxicity studies, regulatory gaps remain, particularly in terms of manufacturing standards, liability, and reimbursement—a challenge that is also evident in the US [23, 24]. A unified regulatory framework is needed to address these gaps and ensure consistent assessments of quality, preclinical, and clinical aspects, including risk–benefit assessment. While the EMA has not provided specific guidance for N-of-1 therapies in the past, the draft guidance on oligonucleotide development and manufacture does begin to address personalized and N-of-1 treatments [25]. However, insufficient guidelines lead to variations in how Member States handle these cases, highlighting the need for more consistent regulation across Europe, particularly regarding quality standards and risk–benefit assessments.

Despite growing advocacy for alternative approaches, such as adaptive pathways and flexible regulatory processes for severely debilitating conditions with limited treatment options, the European Medicines Agency (EMA) has not explicitly moved towards these models in the context of N-of-1 treatments. The EMA’s focus is broader, addressing personalized medicine and advanced therapy medicinal products (ATMPs) rather than N-of-1 treatments specifically. As a result, the regulatory pathways for developing and approving N-of-1 therapies remain complex and are still evolving. Efforts are underway to streamline regulatory processes across Europe. For instance, the Commission Proposal for Pharmaceutical Regulation [26] seeks to establish harmonized procedures for the authorization and supervision of medicinal products across the EU, granting the EMA increased authority. This proposal could help address gaps in areas such as manufacturing and risk assessments while promoting consistency across Member States.

## Strategies and Collaborative Efforts in Implementing N-of-1 and N-of-Few Treatments

Due to varying national and international regulatory procedures, it is crucial to align efforts and share knowledge within the community. In Europe, the absence of a specific regulatory framework for N-of-1 therapies has prompted initiatives like the Dutch Centre for RNA Therapeutics (DCRT) and global collaborations such as the European 1 Mutation 1 Medicine (1M1M) and N = 1 Collaborative (N1C), which aim to facilitate communication and data sharing across borders. Complementary networks, like the European Reference Network for Rare Neurological Diseases (ERN-RND), focus on expertise exchange and patient care.

The success of N-of-1 treatments depends on patient selection, genetic profiling, and tailored ASO treatments. Involving patients ensures treatments align with their needs [27], disease characteristics, and preferences, improving relevance and effectiveness [28].

Academic medical centers can seek regulatory guidance from organizations like the EMA's Innovation Task Force (ITF) to navigate regulatory hurdles. Early engagement with regulators helps developers align with scientific advice and best practices based on previous experiences with non-coding RNAs. Although N-of-1 therapies do not go through a formal approval process by the EMA or European Commission, the DCRT's proactive engagement with the ITF prior to identifying a lead product exemplifies the value of such early interactions. Subsequent meetings involving specific products through ITF and formal EMA-Scientific Advice (SA) within the European 1M1M network highlight the recommendation to seek regulatory guidance throughout the development process. Recently, the DCRT, in collaboration with the N1C, issued global consensus guidelines for individualized ASO development [29, 30], emphasizing best practices dissemination, standardized reference ASOs (creation of an online database of validated control ASOs, a resource the N1C will compile), and continuous data sharing to enhance efficacy and safety. They also proposed leveraging shared data for retrospective analyses and deriving open-source computational design algorithms [29]. Additionally, in partnership with the 1M1M consortium, they published an article outlining their strategy for developing individualized treatments in Europe, with splice modulation emerging as a promising mechanism in ASO therapies for rare neurogenetic disorders [22, 31]. Moreover, the N1C website offers [32]: (1) resources aiding clinicians, scientists, and healthcare professionals in assessing genetic variants for ASO eligibility; (2) chemistry guidelines for ASO development in preclinical animal testing, along with insights into preclinical modeling systems and effective usage tips; and 3) clinical protocol examples provided for reference.

Furthermore, the International Rare Diseases Research Consortium (IRDiRC) initiated a Task Force with the aim to connect different N-of-1 and N-of-few efforts to reduce duplication, achieve global consensus and create a roadmap towards development and implementation of N-of-1 and N-of-few treatments.

Clinicians addressing rare diseases frequently encounter challenges, including limited guidance from health authorities and restricted access to affordable oligonucleotide resources. Collaborative networks can help overcome these obstacles by facilitating the exchange of information, protocols, and resources. Some organizations in the field are dedicated to supporting ultra-rare diseases by providing free therapeutic solutions, though their participation in data-sharing initiatives varies [15]. Nevertheless, opportunities remain for increased collaboration across the sector. [33]

## Case Studies: Successes, Setbacks, and Lessons Learned

In just one year in 2018, the US saw the development of Milasen (Boston Children’s Hospital), a patient-customized ASO, by a Boston-based team for 6-year-old Mila Makovec with neuronal ceroid lipofuscinosis 7 (CLN7), a form of Batten’s disease [34]. The condition was attributed to a unique cryptic splicing variant in the CLN7 gene (Table 1). This pioneering effort paved the way for additional N-of-1 or N-of-few ASOs tailored for individual or a handful of patients using expedited regulatory pathways. [35]

**Table 1:** N-of-1 ASOs (non-exhaustive list).

Drug Name (Market Name)	ROA	Target Gene	Indication	Disease Characteristics	Modality	Chemistry	Mechanism of Action
Milasen	Intrathecal	CLN7 (also known as MSFD8)	Neuronal ceroid lipofuscinosis 7 (CLN7), a form of Batten's disease	Rare, fatal neurodegenerative disease (blindness, ataxia, seizures, developmental regression)	ASO	2′-MOE-PS	Splicing modulation
Valeriasen	Intrathecal	KCNT1	Epilepsy	Severe, highly lethal form of epilepsy (intractable seizures, 40–100/day)	ASO	2′-MOE, gapmer	RNase H1
Atipeksen	Intrathecal	ATM	Ataxia telangiectasia	Rare disorder causing weakened immune function, dilated blood vessels in the eye, skin or mucous membranes, and progressive difficulty with balance, coordination, speech, and eye movements	ASO	2′-MOE-PS	Splicing modulation
Jacifusen/Ulefnersen (formerly known as ION363)	Intrathecal	FUS	Amyotrophic lateral sclerosis	Rare, fatal, neurodegenerative disorder characterized by muscle weakness, loss of movement, and difficulty breathing and swallowing	ASO	2′-MOE-PS	RNase H1
Afinersen	Intrathecal	C9ORF72	Amyotrophic lateral sclerosis	Atypical motor neuron dysfunction	ASO	Mixed backbone 2′-MOE, gapmer	RNase H1

Drug Name (Market Name)	Approval	Institution/Company	Patient(s)	Preclinical results	Results in patient	Side effect	Other patients treated
Milasen	FDA (2018)	Boston Children’s Hospital	Mila Makovec (6 years old)	Restoration of normal splicing and protein production in patient-derived cell lines. Toxicology studies performed in rats	Clear drop in the frequency and duration of epileptic seizures and slower functional decline. However, due to late-stage disease, Milasen could not reverse accumulated neuronal damage. Patient passed away 3 years later in 2021	Acceptable side-effect profile, with no safety concerns	Unique case study
Valeriasen	FDA (2020)	Boston Children’s Hospital	Valerie Schenkel and Lucy Greenblot (toddlers)	N/A	The drug reduced the frequency of seizures in one patient and eliminated them in the other. However, both developed hydrocephalus and stopped treatment. One died from unrelated causes. The FDA has approved plans to restart treatment at a lower dose for one patient	Hydrocephalus	Only 2 patients treated, though treatment was developed as N-of-few treatment for pediatric patients (i.e., tens of such patients are known worldwide)
Atipeksen	FDA (2019)	Boston Children’s Hospital	Ipek (3 years old)	ASO treatment tested on skin cell samples from 3–4 children with similar mutations	Treatment started at an age where signs of neurological impairment did not show yet (typically at age 6–7). Potential to preserve motor function and slow disease progression	No serious adverse effects detected 3 years into the treatment	N/A
Jacifusen/Ulefnersen (formerly known as ION363)	FDA (2019)	Ionis Pharmaceuticals and collaborators at Columbia University	Jaci Hermstad (25 years old)	Drug showed efficacy in FUS-mutant mouse model. Limited toxicity data available but chemically similar to other clinically used Ionis ASOs for ALS	Patient died of complications of the disease 10 months after the first injection (a total of 12 infusions, each 20–120 mg). Neuropathological examination showed that ION363 reduced wild-type and mutant FUS protein with a decrease in FUS-containing aggregates. There was little nuclear FUS staining in the spinal cord and motor cortex, and FUS-containing aggregates in motor neurons were reduced compared with the untreated ALS-FUSP525L control, in which FUS aggregates were abundant	No serious adverse effects detected	12 INDs submitted in total for this therapeutic; which led Ionis to sponsor a proper clinical trial initiated with up to 95 participants (≥ 10 years old)
Afinersen	FDA (2019)	Collaboration between the RNA Therapeutics Institute at UMass Chan Medical School and several foundations	A 60-year-old man	Testing in mice, sheep, and cynomolgus monkeys	CSF polyGP levels decreased by 80%, indicating ASO activity and reduction of C9orf72 expansion consequences. ALSFRSR remained stable throughout treatment	N/A	Potentially applicable to other patients with the same mutation

For instance, Atipeksen (Boston Children’s Hospital), an individualized ASO designed for a specific mutation causing ataxia-telangiectasia (AT) [36], initiated at an early age in a 3-year-old patient. Notably, this treatment started before the typical onset of neurological symptoms (typically at age 6 or 7), showcasing the potential to preserve motor function effectively.

Furthermore, the development of Afinersen (RNA Therapeutics Institute, UMass Chan Medical School, Massachusetts) targeting Amyotrophic Lateral Sclerosis (ALS) with a C9ORF72 mutation underwent a single-patient pilot study in 2019 in a 60-year-old man, following successful animal model testing. This suggests potential applicability to other patients with the same gene mutation [37]. Jacifusen (Ionis Pharmaceuticals, California), an ASO tailored for a particular mutation in FUS causing ALS, demonstrated promising efficacy in preclinical studies and subsequent patient applications [38]. Jaci Hermstad, aged 25, was the pioneering recipient of this treatment, diagnosed in February 2019, coinciding with the ongoing development of Jacifusen by Ionis (formerly known as ION363). Subsequent to Jaci's treatment, numerous other patients received the therapy, with approximately 12 IND applications submitted for the same therapeutic. This substantial interest culminated in a comprehensive clinical trial sponsored by Ionis (NCT04768972), marking a pivotal shift from individual case studies to broader clinical applications. [39]

Currently, many N-of-1 treatments are focused on brain and eye diseases (Table 1) due to the relative accessibility of these tissues and the challenges associated with targeting other tissues, such as muscles. While Table 1 provides examples of these innovative approaches, it is important to note that this is not an exhaustive list, as numerous other treatments and programs are also underway, reflecting the breadth of advancements in the field.

However, alongside these success stories, setbacks have been encountered, as illustrated by the ASO Valeriasen (Boston Children’s Hospital), underscoring the complexities inherent in personalized treatments. In 2020, two toddlers with a severe form of epilepsy, both harboring a KCNT1 mutation, were treated with the N-of-few therapy. While the drug reduced seizures in one patient and eliminated them in the other, both developed hydrocephalus, resulting in the cessation of treatment [40]. Although one patient's death was unrelated, the trial was paused, pending FDA approval to resume therapy for the second patient at a lower dose, with investigations underway to mitigate hydrocephalus risk. Similar occurrences of hydrocephalus have been noted with ASOs for other conditions, such as Tominersen (Genentech, California) for Huntington’s disease [41] and Nusinersen (Biogen, USA) for spinal muscular atrophy [42], although establishing causation proves challenging due to the multifaceted nature of neurologic diseases and varying ASO characteristics. Such setbacks emphasize the importance of thorough risk assessment and continuous monitoring in the pursuit of personalized therapies.

In Europe, ASO examples include, among others, ongoing trials for PLP1 mutation-induced hypomyelination of early myelinating structures and trials for Atipeksen, and the development of an individualized therapy by Institut Imagine for a patient with a KCNB1 gene mutation [43], highlighting the global momentum in advancing tailored treatments for rare genetic mutations. [44, 45]

In addition to ASO-based approaches, CRISPR gene editing has emerged as a potential N-of-1 and N-of-few therapy. CRISPR technology, unlike RNA-based therapies, directly edits the DNA to correct genetic mutations, offering a more permanent solution. A notable example is CRD-TMH-001 developed by Cure Rare Disease (Boston, Massachusetts) for Duchenne muscular dystrophy (DMD) [46]. This therapy, targeting muscle promoter and exon 1 mutations on the dystrophin gene, entered a clinical trial (NCT05514249) in 2022 for a patient with DMD. Unfortunately, the patient's health deteriorated rapidly six days after receiving the therapy, displaying cardiac and respiratory distress that led to their death. Post-mortem findings revealed lung injury due to a heightened immune response to the high-dose AAV vector [47]. The absence of Cas9 expression in the patient's body indicated the therapy remained inactive. However, this highlighted the potential strong toxic effect of 'first generation' vectors like AAV9 when delivered at high doses needed for widespread reach to muscles, particularly in patients at advanced disease stages with weakened physical conditions.

The main challenge in N-of-1 CRISPR cures is the irreversible nature of gene editing compared to the adjustability of ASO dosing. Balancing accelerated de-risking and patient safety is crucial. While significant investments can be justified for chronic severe diseases with large patient pools reaching hundreds of thousands, the exorbitant costs (up to $5 million) associated with N-of-1 CRISPR for unique cases, where the window for life-saving editing is narrow, render this approach currently impractical.

## Challenges and Future Directions

Despite promising therapeutic achievements, the development of these therapies for ultra- and nano-rare diseases faces major hurdles.

One major difficulty is the inadequacy of the conventional regulatory drug development path for N-of-1 or N-of-few trials. Placebo-controlled trials, a cornerstone of conventional drug development, become impractical for ultra-rare diseases due to the limited number of patients available for such trials. Moreover, the individualized nature of these therapies introduces complexities in standardizing outcome measures for safety and efficacy testing, further hindering the standardization process across treatments. Additionally, the absence of a clinical development track record for specific rare diseases complicates trial design and optimal outcome measure selection, necessitating substantial time and financial investment. Clinicians often draw analogies to more prevalent diseases with similar manifestations to guide endpoint selection, adding another layer of complexity.

Furthermore, the high heterogeneity of ultra-rare diseases presents a unique challenge, often requiring specific primary endpoints tailored to each patient in a small clinical trial. For instance, in a Phase III trial (NCT02110147) of Xuriden (Wellstat Therapeutics, USA) [48], a treatment for hereditary orotic aciduria, each of the four patients had different primary endpoints based on their particular hematological manifestations, illustrating the need for personalized approaches even within small patient cohorts.

Moreover, uncertainties persist regarding the long-term efficacy and risks of innovative therapeutic approaches lacking a proven track record. Financial burdens associated with rare disease therapy development further exacerbate the challenges, as restricted patient eligibility drives up treatment costs per patient despite comparable costs to common disease therapies [24]. For instance, the development of investigational ASOs like Milasen and Atipeksen required backing from charitable foundations and cost an average of $1.6 million per program [35]. These costs raise concerns about the scalability and the ethics of dedicating significant resources to treatments for very small numbers of patients, even though they are comparable to the costs of other genetically targeted therapies like Nusinersen or Zolgensma (Novartis, Switzerland) (an ASO and a gene therapy used to treat spinal muscular atrophy, respectively).

Targeting specific tissue and cell types, such as muscles, remains a difficult task despite advancements in chemical modifications. Repeated dosing through invasive procedures, especially for intrathecal delivery, poses additional complications. However, advancements in chemical modifications aim to enhance metabolic stability, reduce dosing frequency, and improve delivery across biological barriers. For example, researchers are developing cell-penetrating peptides and ligands to improve tissue targeting and cross the blood–brain barrier, promising advancements in targeted delivery methods [49].

Moreover, exon skipping therapies face specific hurdles, including underdiagnosis of non-coding variants, inadequacies in current splicing tools, incomplete understanding of variant effects, and uncertainties in in vivo dosage sensitivity, which impact both prenatal and postnatal functions.

In addressing these challenges and charting future directions, artificial intelligence (AI) emerges as a promising tool [50]. AI could expedite the clinical development of ASOs for N-of-1 and N-of-few by analyzing patient data to pinpoint individuals with targetable genetic diseases. Additionally, AI may aid in swiftly identifying promising target sites and predicting optimal positioning for splice-shifting oligonucleotides, thereby reducing the number of compounds needing screening and accelerating the discovery of new ASOs [51, 52]. Collaborative efforts among the pharmaceutical industry, academia, and federal agencies are crucial in leveraging AI's potential, along with establishing a federated, decentralized patient database to facilitate data sharing and analysis for personalized therapies.

## Toward a Specified Design Envelope for Personalized RNA Therapeutics

A design envelope in the field of medical devices refers to the set of design parameters within which a patient-matched device can be produced to meet the specific needs of an individual patient. It includes boundaries for variations in device design to ensure consistent performance and safety [53]. This concept is critical in providing guidance on verification and validation aspects of patient-matched medical devices and medical device production systems (MDPS), promoting a harmonized regulatory approach.

**Figure 1:**
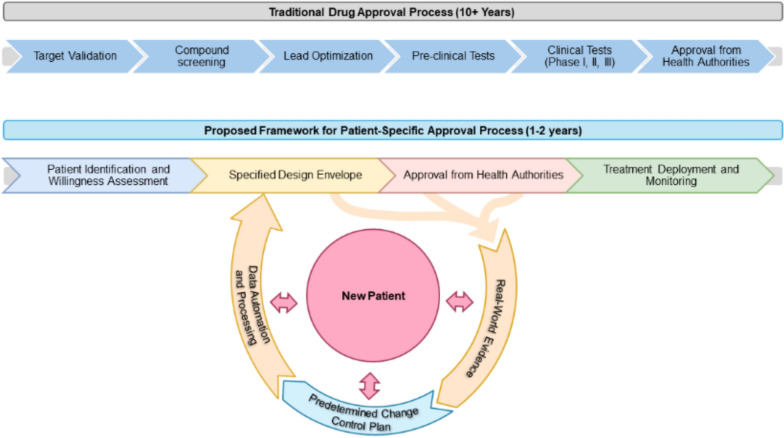
Streamlined Patient-Specific RNA Therapeutics Approval Framework: A Comparative Analysis with the Traditional Drug Approval Process.

**Figure 2:**
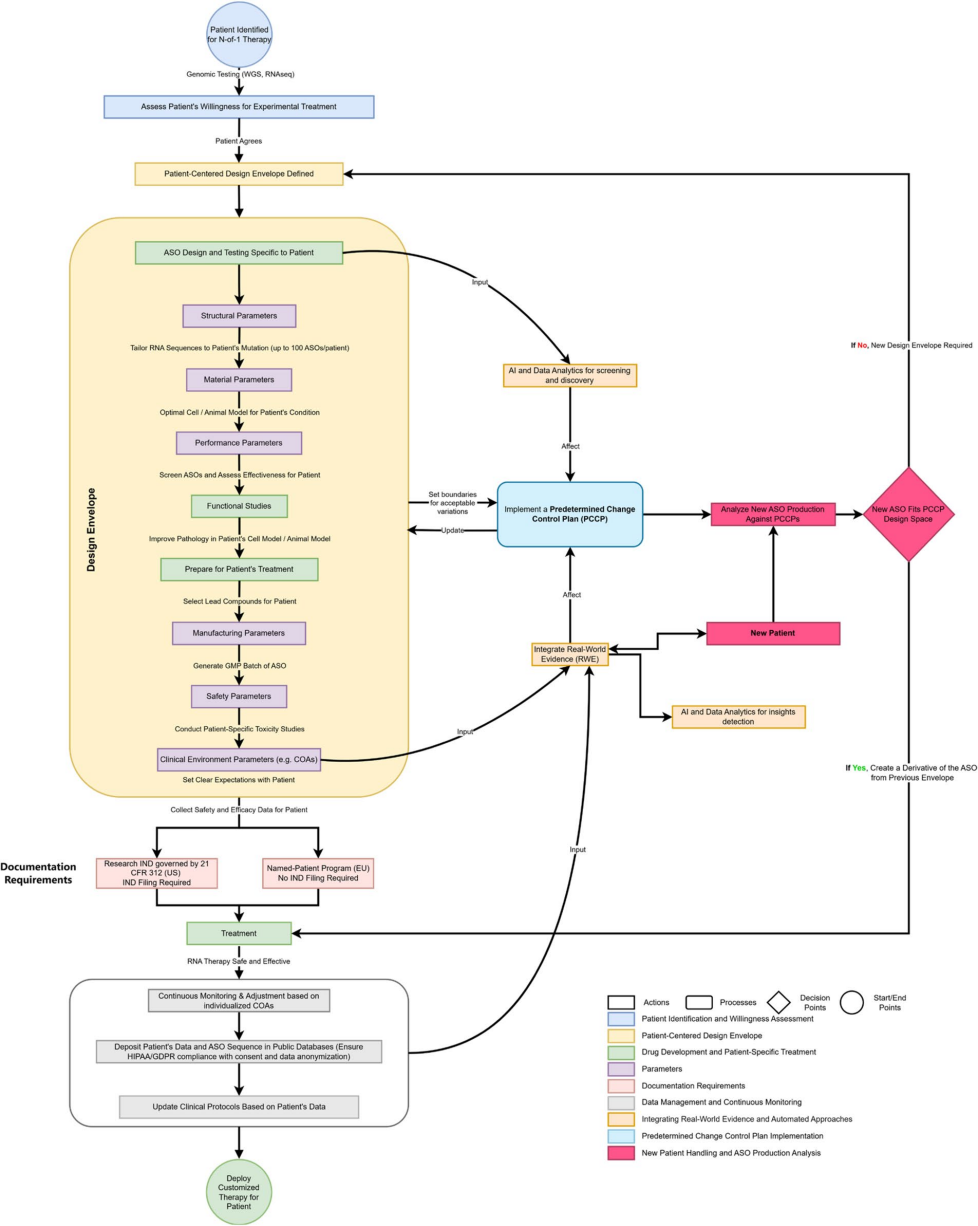
RNA Therapeutics Design Framework and Predetermined Change Control Plan (PCCP). This comprehensive framework outlines the step-by-step process for designing, testing, and implementing N-of-1 and N-of-few RNA therapeutics for personalized patient care. It incorporates patient-centered design principles, continuous monitoring, real-world evidence integration, and the use of AI for improving treatment outcomes. The predetermined change control plan ensures that any changes in the ASO production are managed systematically, maintaining the integrity and safety of the therapeutic process. This framework is only a proposal and is subject to further investigations and approval from stakeholders and health authorities.

The nascent regulatory landscape governing N-of-1 and N-of-few treatments, highlights the need for a structured framework to ensure safety, efficacy, and patient welfare. Drawing inspiration from established norms in medical device development [53], we propose a comprehensive framework (Figs. 1 and 2) based on the definition of a specified (and openly disclosed) design envelope for the range and type of N-of-1 and N-of-few therapies developers seek approval to produce. This design envelope, characterized by multidimensional parameters detailed in Table 2, describes the entire treatment lifecycle—from design and manufacturing to clinical execution—underpinned by AI-driven analytics, automation and real-time data integration to enhance safety and responsiveness to patient needs.

**Table 2:** Design envelope parameters

Parameter Type	Description
Structural Parameters	Tailoring RNA molecule sequences to patient-specific genetic variations and disease profiles, optimizing RNA length for target engagement, and incorporating modifications to enhance stability, specificity, and delivery
Material Parameters	Ensuring high purity and integrity of RNA molecules, free from contaminants or impurities, is essential for therapeutic effectiveness. Additionally, selecting appropriate delivery systems, such as lipid nanoparticles or viral vectors, is paramount for efficient targeting of cells and tissues, thereby maximizing therapeutic efficacy
Manufacturing Parameters	Consistent and reproducible production of RNA therapeutics is paramount. Optimized synthesis methods and formulation strategies ensure stability, bioavailability, and safety during storage and administration, thereby enhancing treatment efficacy and patient adherence
Performance Parameters	Monitoring target engagement and assessing therapy efficacy beyond traditional measures are essential for optimizing treatment outcomes. Parameters like splicing ASO treatment on secreted proteins, as well as potential lifelong effects including drug resistance and dosing effects, inform treatment optimization and patient monitoring strategies
Safety Parameters	Assessment and management of immune responses, as well as rigorous screening for impurities and off-target effects, are paramount for ensuring therapy safety. By delineating core side effects from those specific to the disease being treated, clinicians can effectively balance benefit and risk, establishing thresholds for decision-making based on these factors
Clinical Environment Parameters	Integration of real-world evidence (RWE) and clinical outcome assessments (COAs) into the therapy design process provides dynamic feedback and continuous refinement, allowing clinicians and regulators to gather invaluable insights into treatment efficacy, safety profiles, and long-term outcomes. Decision-making processes are thus enriched by incorporating patient-specific factors, including genetic variations and disease progression, into therapy design and monitoring

Our proposed framework is sufficiently flexible to accommodate the unique attributes of RNA-based therapies, such as nucleic acid stability and delivery mechanisms. The design envelope could be assessed by regulators alongside initial N-of-1 and N-of-few treatments authorization, streamlining the approval process for the manufacturer’s quality management processes throughout the therapy lifecycle. Additionally, it could provide a priori approval for extending from the first ‘sentinel’ therapy to the fast-track approval of subsequent therapies, if they fall within the predefined scope (i.e. the approval envelope). AI algorithms assist in optimizing these parameters within the design envelope, enabling more efficient production, monitoring, and validation processes.

The FDA's Platform Technology Designation Program (introduced in May 2024) [54] and the EU's draft Pharmaceutical Legislation (Article 15) [55] propose regulatory mechanisms to streamline the approval of therapies based on shared technology platforms. These initiatives aim to facilitate and potentially expedite the approval process for treatments that leverage pre-existing platform-based authorizations, thereby maintaining a focus on safety and efficacy. Although they do not explicitly reference or address N-of-1 therapies in their current form, they could indirectly benefit the regulatory processes for N-of-1 to N-of-few therapies in the future.

## Integration of the Predetermined Change Control Plan (PCCP) for RNA Therapeutics

In line with the FDA’s 2024 guidance on PCCPs, we integrate the Predetermined Change Control Plan (PCCP) [56, 57] concept, originally designed for Medical Devices, into the context of highly personalized RNA therapeutics. The PCCP allows for predefined changes to be made within an approved design envelope, avoiding the need for a new marketing submission for each modification, provided these changes remain within the pre-approved scope of the plan. This approach enables a structured and efficient pathway from N-of-1 to N-of-few treatments, and from one N-of-1 treatment to highly related N-of-1 treatments for similar conditions. It will ensure that pre-approved changes can be safely and efficiently implemented.

In the context of RNA therapeutics, the PCCP provides a framework for managing changes by implementing structured procedures to anticipate, assess, and adapt to modifications in therapy design (e.g., RNA sequence), manufacturing processes, or clinical protocols. AI and automation play a pivotal role in this process by predicting potential modifications in therapy design and optimizing manufacturing workflows. AI-powered real-world evidence monitoring further enhances this framework by continuously collecting and analyzing patient data, enabling real-time adjustments to treatments without compromising regulatory compliance. This ensures continued efficacy and safety, facilitating the rapid and efficient development of new RNA-based treatments as needed. By aligning with the FDA's guidelines on PCCPs, this approach can support the continued innovation of RNA therapeutics while maintaining the highest standards of quality, safety, and regulatory compliance.

## Specificity and Pre-approval of Changes in RNA Therapies

One of the key elements of the FDA’s PCCP guidance is that modifications must be specific and predefined. If we transpose this to RNA therapies, this means that any changes in the RNA sequence, delivery vectors, or production methods must be planned in advance and clearly outlined within the PCCP. The FDA recommends that each PCCP contain a detailed description of the specific modifications, including the testing, verification, and validation processes that will ensure safety and efficacy. The PCCP will also include an Impact Assessment to evaluate how these modifications may affect patients or the overall therapeutic profile.

Moreover, modifications under the PCCP must be traceable, ensuring that every change aligns with the predefined specifications. This approach ensures that RNA therapies remain safe and effective even as necessary adjustments are made to the treatment over time. The goal is to avoid the need for continuous FDA resubmissions, which would delay critical treatments, while still maintaining a stringent regulatory oversight process.

## Current Regulatory Applicability in the FDA and EU Contexts

As of today, this model, supported by the PCCP framework, could be applied within the FDA regulatory system, as an IND submission is required for N-of-1 treatments. While the concept of PCCPs is still relatively new, and FDA officials are in the process of determining the full scope of changes that can be allowed within a premarket submission, the framework provides a potential pathway for pre-approving modifications. For initial N-of-1 RNA therapies, an IND submission will still be required. However, subsequent modifications that fall within the approved PCCP can be implemented without the need for additional marketing applications. These modifications are considered pre-approved, ensuring that therapies can be rapidly adapted to evolving clinical needs while maintaining a focus on safety.

However, this model is currently a projection for the EU, where the regulatory approach for N-of-1 treatments differs. In the EU, an IND submission is not required for N-of-1 therapies. Instead, the responsibility for such treatments falls primarily on the treating physician, who initiates and oversees the treatment process. This means that while the PCCP framework could theoretically provide similar benefits in the EU, its implementation would depend on future regulatory developments and the willingness of healthcare providers and regulatory bodies to adopt such structured, pre-approved change management processes.

## Benefits of a Specified Design Envelope with PCCP

The concepts outlined within the design envelope and the FDA’s PCCP framework serve as a benchmark ensuring comprehensive consideration of all production aspects for N-of-1 treatments. By integrating the PCCP, we offer an approach that accelerates approval timelines while upholding rigorous safety and efficacy standards of N-of-1 and N-of-few therapies. The flexibility afforded by the PCCP framework ensures that the development, validation, and implementation of RNA-based therapies can proceed smoothly, accommodating necessary changes while avoiding unnecessary delays. Alongside these concepts, the adoption of automated approaches and AI-based predictive models for data analysis and therapy optimization can further enhance the efficiency of the design envelope. These methods can enhance the efficiency and effectiveness of personalized RNA therapeutics. [50–52]

By adapting the PCCP concept from medical devices to RNA therapeutics and leveraging the power of AI, this framework can provide a structured approach to managing changes in highly personalized treatment modalities, ensuring safety, efficacy, and regulatory compliance while facilitating innovation and patient-specific adjustments. This ensures that as new RNA-based treatments are required, they can be quickly and efficiently developed, meeting the urgent needs of patients while maintaining rigorous standards of quality and safety.

## Conclusion

The emergence of personalized RNA therapeutics marks a transformative development in the potential for tailored treatment of individuals with rare diseases. N-of-1 trials, rooted in open science principles, offer a pathway to tailored treatments while fostering inclusivity and diversity in research. Embracing these principles could allow personalized RNA therapeutics to revolutionize patient care for rare genetic disorders. ASOs currently show most promise as N-of-1 treatments, particularly in brain and eye diseases, where they have potential for mutation-specific interventions. While this approach holds promise, it is accompanied by a spectrum of challenges, from regulatory hurdles to complexities in trial design and therapy development.

Addressing these challenges necessitates global collaboration, transparency, and active involvement of stakeholders to streamline development and regulatory procedures, ensuring equitable access to individualized therapies. Persistent issues surrounding payment and sustainability underscore the need for ongoing dialogue and innovative solutions. Additionally, exploring the potential of structurally similar ASOs, though not exact copies, raises intriguing questions about data utilization and trial dynamics, amidst varying trial circumstances and regulatory scrutiny.

To tackle these complexities, we propose a structured framework encompassing multidimensional parameters, leveraging real-world evidence and AI-driven approaches [58, 59]. Our proposed extensible framework for N-of-1 to N-of-few personalized RNA therapy design provides a conceptual roadmap for navigating these challenges. However, further investigation into the intricacies of this framework, particularly focusing on predetermined change control plans, will be essential, forming the focus of forthcoming research endeavors and stakeholders’ validation.

## Data Availability

No datasets were generated or analysed during the current study.
